# The impact of TV series consumption on cultural knowledge: An empirical study based on gratification–cultivation theory

**DOI:** 10.3389/fpsyg.2022.1061850

**Published:** 2022-12-22

**Authors:** Tanin Tirasawasdichai, Bojan Obrenovic, Hussain Zaid H. Alsharif

**Affiliations:** ^1^School of Media and Communication, Shanghai Jiao Tong University, Shanghai, China; ^2^Faculty of Liberal Arts and Management Science, Kasetsart University, Chalermphrakiat Sakon Nakhon, Thailand; ^3^Zagreb School of Economics and Management, Zagreb, Croatia; ^4^Luxembourg School of Business, Luxembourg, Luxembourg; ^5^Business School of Hunan University, Changsha, China

**Keywords:** cultural product, Chinese TV series, cultural acceptance, cultural awareness, cultural knowledge, gratification-cultivation theory

## Abstract

This study aims to clarify the media-induced trends of cross-cultural transmission and examine the implicit promotional potential for cultural branding. The gratification and cultivation theories are used to explore the promotional media prospect in forming perceptions of foreign cultures’ traditions, habits, norms, and values to contribute to international communication. We analyzed the theoretical applicability in the case of China–Thailand contemporary media culture. A total of 856 Chinese series watchers were surveyed. Structural equation modeling was used to analyze the path effect of consumption of Chinese TV series on other endogenous variables. Results showed that cross-cultural media product consumption strengthens bilateral relations. Moreover, the acceptance and appropriation during engagement with media characters and producers lead to favorable attitudes toward the target culture. Results confirm the positive mutual association between the gratification and cultivation theories and their applicability in the current context. This study offers an important contribution through its finding that the need for gratification significantly and positively impacts consumption and cross-cultural learning and raises cross-cultural awareness, thereby leading to sustainable practices.

## Introduction

Cultural products are tangible and intangible assets that include performing and visual arts, heritage conversation, and content media (Aiello, 2014). The technological development of media and communication increases cultural product consumption as it facilitates media access *via* online platforms (Siddharthan and Narayanan, 2018; Pacheco, 2020). Advanced technology and methods are also used for semantic reasoning (Zheng et al., 2021a,b, 2022a). Natural language inference (Zheng et al., 2022b) and extracting core information from images and texts (Zheng et al., 2021c) are conducive to mapping knowledge, which is especially useful in media interpretation. Knowledge Foreign consumers are considered potential promotional targets internationally, and brands and destination managers are increasingly collaborating with producers to capitalize on these consumers’ interests (Mendes et al., 2017). Aside from entertainment and educational purposes, the export of cultural products such as tv shows and movies, facilitated by online access, can significantly boost the domestic economy and create a favorable climate for international trade, employment and tourism (Lee, 2019), increasing the income from fashion, entertainment, food and tours. Thus, with widespread and easy access through digital channels, cultural product expansion has increased significantly (Wei and Rafael, 2021). The concept of this issue has been quite popular over last decades as creativeness not only brings financial added value, but also in the non-monetary sense it contributes to establishment of soft power (McClory, 2016). Scholars even pay more attention to soft power, which is defined as cultural power as well, rather than hard power, since they believe that soft power could convince people from inside by their mind without using any coercive or financial tools (Nye, 2021). The cultural sector can promote economic growth through different activities and across industries (e.g., export expansion through sales in tourism, creative industries, fashion, and food). Hence, culture is a key factor for country development in several dimensions. Chinese TV series played a key role in the development of cross-cultural product consumption among Thai people, particularly *via* Chinese online applications, and consequently increased diffussion of other cultural, traditional and lyfestle products and services. Return on the national creative and tourist industry is immense, and subsequent consumption will additionally boost GDP and national economy. Acceptance and appropriation in the identification process and engagement with media content led to favorable attitudes toward the target culture, ultimately reinforcing positive convictions and attitudes regarding the culture in question.

To enter global trading of the cultural products, China has rigorously sought the advancement of communication channels (Keane, 2019). Over a billion internet users globally access Chinese applications *via* online platforms to consume Chinese TV series (Gilardi et al., 2018). In Thailand, Chinese TV series are popular among internet consumers. Thai people spend about 3 h and 44 min using online media, with 98% of this time dedicated to online videos (Chankisean, 2020). Tencent Video, one of the largest online video platforms, prioritized the Thailand market to expand its service through We TV, an online video application that grew by more than 700% in less than 2 years (Branded Content, 2020). The growing popularity of Chinese TV series serves as a diffusion mechanism for the transmission of cultural values, ideas, attitudes, and perspectives.

Thailand has a large population of Thai Chinese that migrated from China more than a thousand years ago (Prattanasanti, 2019). Chinese culture has undoubtedly been crucial in shaping Thai culture for centuries (Jiang, 1966). With the increasing dissemination of cultural products, Chinese culture has become increasingly influential (Han, 2021). Chinese cultural product consumption is pivotal in foreign consumers’ cultural learning through the promotion of the country’s image, reputation, and status, as well as the diplomacy arising through the nurturing of favorable relations (Guo et al., 2012). With the appropriate and comprehensive understanding of media consumption and drivers of the cross-cultural learning process, authorities could propose a sustainable international communication policy.

Culture is the culmination of societal historical achievements which can be generationally passed down, and as such, it encompasses enduring behavioral norms, values and attitudes (Matsumoto and Juang, 2007). According to Hofstede et al. (2010), cultural dissimilarities can be identified through a layer-like set of diverse characteristics. Based on such a characterization, we can derive a concept of a ‘foreign’ culture as that which diverges from another society due to its particular symbols, including verbal and non-verbal language and mannerisms, its unique national heroes, historical or current otherwise especially admired and esteemed by ethnicity, and set of moral and behavioral values that are homogenously pursued across the community. Even though Thailand and China are seemingly quite similar to each other, the Chinese language is still not as popular or mandatory as English (Rojanaphruk, 2022).

Thai and Chinese cultures use different contextual metaphors, metonymy, euphemisms and rhetorical methods to express and identify feelings, attitudes and emotions. Furthermore, there are significant clashes in their respective political systems and religious beliefs, lifestyles, cuisine, and celebrities (Duan, 2020). Trompenaars and Hampden-Turner (2002) argue culture constitutes three layers – the outer layer consists of language, buildings, art, fashion and cuisine, the middle layer consists of peculiar norms and values and the core layer concerning basic existential assumptions. Following provided classification, despite the many implicit resemblances between Thai and Chinese citizens, Chinese culture is legitimately both conceptually and tangibly foreign with respect to Thailand’s value hierarchy, linguistic background and moral sensibilities. Furthermore, Chinese cultural products significantly intensify cross-cultural contact by increasing Thai citizens’ understanding and acceptance of Chinese culture and act as facilitators of cross-cultural competencies, traditional assimilation and boost international relations (Spitzberg and Changnon, 2009; Budzanowska-Drzewiecka et al., 2016).

To develop a functional research framework, we integrate the gratification and cultivation theories in this study. Cultivation theory effect has been linked to attitudes in prior studies (Arendt, 2010) and to explain social media impact on behavior formation (Tsoy et al., 2021, 2022). In this study we combine the uses and gratification theory (U&G) and cultivation theory (Tapper, 1995; Minnebo, 2000). The integration of these two theoretical frameworks promotes our understanding of the effects of media consumption and cross-cultural learning. In other words, this combination could provide a clearer picture of how theoretical processes work (Bilandzic and Rössler, 2004). Nevertheless, to the best of our knowledge, studies on gratification–cultivation theory are scarce despite being regarded as a core theory in the media and communication field.

Culture is a key role in many significant aspects of development. Previous studies focused on culture as an intangible asset or resource for increasing the tourism industry’s attractiveness (Fainstein et al., 2003). Furthermore, some authors uniquely emphasize distinct cultures through the very presence of the cultural industry which has been turned into a creative industry or creative economy. Cultural promotion and branding through the entertainment industry are relatively established and widely researched phenomena, especially among Western nations. Although online media promotional and advertising potential and drivers of consumption are fairly familiar to governors, marketers and international relations specialists and vast evidence on the effectiveness of marketing and management strategies is generated daily, explanatory methodological frameworks deployed therein are strictly applicable to English-speaking, highly individualist and capitalist societies. However, such groundwork may not be suitable in collectivist, reserved and closed-off societies and there is an evident lack of empirical explanation of cross-cultural product consumption and branding through media advertising among Asian societies. To the best of our knowledge, this is the first study of its kind concerning Chinese-Thai cultural product consumption. The current paper presents a preliminary attempt to broaden our knowledge of cultural branding in collectivist societies. We seek to evaluate the pertinence and usefulness of Anglophonic U&G and Cultivation frameworks in Asian communities.

The complementary objective is to shed light on media-induced trends of cross-cultural transmission and examine the implicit promotional potential for product and destination branding. The applicability of Gratification Cultivation theory as the basic conceptual framework through cultural product consumption is also our objective to provide the characterization of Cultural knowledge and cross-cultural consumption between China and Thailand. As aforementioned although there is a closeness between these two countries, adapting the main theoretical Western origin model could provide a wider context application of cultural dissimilarities through cross-cultural consumption.

All these concepts and backgrounds bring to the fort research questions to fulfill this gap in cultural knowledge and learning through cross-cultural consumption. For example, as individuals consume cultural products, does the gratification impact their cultural learning? How does cultural product consumption affect cross-cultural acceptance and knowledge and how it contributes to other aspects of development?

The rest of the paper is structured as follows: The theoretical background and literature review, serving as the basis of the research hypotheses, are presented. Then, the methodology is described, and the findings and discussion are provided. Finally, the limitations and future directions are proposed.

## Theoretical background

Our theoretical framework is expected to account for how the learning of a dissimilar culture results from the cultural acceptance, which can be predicted by enjoyment and narrative engagement (Rubenking and Bracken, 2018). Gratification stands as an antecedent and a motivational driver for viewer engagement while the cultivation effect stems from several parallel processes (Soto-Sanfiel et al., 2021). The engagement component of gratification affects the social construction of mediated reality, and the perceived reality is the result of the cultivation effect (Busselle and Bilandzic, 2009). What is being cultivated through cinematic experience is primarily the consumption of cultural products (e.g., TV series), out of which producing nations cultivate cultural and sociopsychological awareness, favorable attitudes, and the desired behavior (Fu et al., 2020). The outcome, that is, the cultivated behavior, can range from cultural acceptance to an increase in consumption of other industrial products (e.g., tours, fashion, food, and technology).

Gratification–cultivation theory, which combines the U&G and cultivation theories, is adopted as the theoretical framework of this study. Although the two theories seemingly differ in theoretical directions, examining them under the scene of cultural digital product consumption clarifies their association. U&G theory explores people’s demand in media usage, the objectives and satisfaction levels of which differ from those of media consumption (Dhir et al., 2017). Such demand is supposed to fulfill users’ expectations and gratifications. Various media types provide different or specific features depending on which media aspects can serve users’ needs (Katz, 1959).

Gratification could go beyond the entertainment and relaxation purposes of media use. For example, Rubin (1983) stated that some people watch TV programs to avoid escapism and stay informed rather than seek enjoyment. In the online fandom community, fans socialize regarding content consumption while encouraging the participation of others, thus resulting in high consumer involvement (Jenkins, 2007; Fiske, 2012). Papacharissi (2009a) mentioned that people consume media to meet their demands, wants, or desires. Apart from amusement, information search, communication with other consumers, and participation in digital or online communities, individuals use media for self-expression (Choi et al., 2015). New media platforms enable the global broadcast of TV content (Pacheco, 2020; Choi et al., 2021), thus transcending physical boundaries and linguistic differences and increasing cultural diplomacy and cultural promotion (Jin, 2020).

Cultivation theory was initially introduced to account for how media consumption im-pacts consumers’ world views (Gerbner and Gross, 1976). At that time, television was the primary media source. As technology developed, consumers could access media conveniently and spend more time absorbing content (Park et al., 2014). Gerbner et al. (2002) stated that intensive viewers tend to believe that their lives are similar to those they see on TV. Many previous studies have confirmed parasocial interaction, in which identification with fictional characters shape audiences’ attitudes, viewpoints, behaviors, etc. (Madison and Porter, 2016; Aytulun and Sunai, 2020; Javed et al., 2022). Viewers identify media characters as similar to themselves or others close to them (Cohen, 2009; Tsoy et al., 2021). The management of a narrative and reinforcement of mediated perception of a foreign culture is explained according to cultivation theory. The theory accounts for how the integration of cinematic and cultural context portrayed in particular content is to be interpreted by consumers (Lovric, 2018). This in turn spikes viewers’ interest and prompts audiences to further explore the culture of their favorite characters and their respective histories (Lee, 2019; Javed et al., 2022).

The two theories seemingly differ in their theoretical directions, but their association can be clarified by examining them in the modern media landscape fueled by the need for instantaneous gratification and motivation for consumption in the context of globalization. They relate to each other in parasocial interaction and knowledge acquisition, especially in the recent collaboration between the advertising industry and production, that is, the globalization trend of TV fashion. Parasocial interaction, that is, the identification with fictional characters, has been confirmed to be related to changes in audiences’ attitudes, habits, and behaviors (Madison and Porter, 2016; Aytulun and Sunai, 2020; Javed et al., 2022).

Prior studies have indicated that media exposure results from motivations and not from beliefs (Bilandzic and Rössler, 2004). Although this concept may not be valid in every cultivation measure and genre, Tapper (1995) confirmed that motivations are crucial in analyzing and explaining cultivation in television use. Motivation and cultivation effects thus overlap, and some researchers have tried to follow the U&G theoretical concept while using the dataset of cultivation theory synchronously (Minnebo, 2000). The motives in media exposure are indicated as the initiation, which later impacts users. Bilandzic and Rössler (2004) identified that cultivation is initiated with the gratification sought by viewers from television. Thus, U&G could be regarded as a potential feature in television programs. Al-Shaqsi (2000) argued about the dynamic relationship between audiences, messages, and effects of media consumption. Therefore, U&G should be included in the investigation of cultivation effects.

The two theories are crucial to explaining viewers’ attitudes and perceptions (Atkin et al., 1983). Regardless of the contradiction between these two theories among active and passive viewers, they appear to complement each other. U&G theory focuses on the psychological need from media use, whereas cultivation theory focuses on how media shapes users’ attitudes and perceptions. Thus, the combination of these theories could explain how the theories work.

Understanding the process of cultural learning from foreign media consumption plays a very crucial role in international communication policy authorities. Especially, in era of media volatility among globalization and diversity, the good comprehension would bring to the appropriateness of international media and communication manners.

## Research model development

### TV series consumption

To understand the motivation and gratification in TV series viewing, this study applied U&G theory. The theory was used to identify the motivations behind media use (Papacharissi, 2009b). Technological development has changed consumption processes, as well as consumers’ gratification gained and sought (Eighmey and McCord, 1998). Currently, consumption is greater through online digital platforms than through traditional media. The instantaneous on-demand content availability could shape consumers’ views, values, and attitudes.

The concepts and values consumers associate with certain cultures are modified and reinforced by audiovisual products, such as popular and engaging TV shows and movies that highlight inherent culture through narratives or character identification (Lee, 2019; Vila et al., 2021). Cultivation theory explains how mediated reality may cultivate perception and even lead to identification. One of the fundamental drivers upon which viewers are incentivized to consume the content of cross-cultural products is the need for gratification. Consumers receive gratification through the very act of consumption that extends from spectators’ need for entertainment and excitement. Meanwhile, gratification can arise from viewers’ engagement, feeding into the need for socialization based on common interests, as in the case of fandom communities. Therefore, cultural awareness resulting from consuming cross-cultural products increases with the productions’ ability to convey essence and heritage through narrative stories (Pacheco, 2020). Characters and places portrayed can differ significantly from viewers’ personal backgrounds, in which case audiences become incentivized to learn more of the foreign folklore or even visit filming locations (Vila et al., 2021). The consumption of the content of individual TV programs results in enjoyment (Stafford et al., 2004), as in the case of TV series watching among college students (Huang et al., 2015). Gratification sought and obtained could also stem from the new functions of technological tools (Sun and Zhong, 2020), applications, operating systems in gadgets, and internet availability. Luo and Remus (2014) suggested that U&G is the fundamental theoretical framework of cultural product consumptions, specifically in the context of the diversity of media technology and content. Audiovisual media has been found to play a massive role in contemporary visual centricity (Nicolaou and Kalliris, 2020).

Thus, we hypothesize the following:

*H1*: The gratification sought by consumers positively influences TV series consumption.

*H2*: TV series consumption positively influences the gratification obtained.

### TV series consumption and cultural awareness

Cultural awareness is the first step in the acknowledgment of cultural product consumption. Nicolaou (2021) found that audiovisual media supports learning through streaming and that the depicted international genealogical characteristics and habits, culturally inherent characteristics, and sociocultural identity can influence knowledge acquisition. Cultural awareness is how one can perceive values of culture and cultural habits that are dissimilar to himself/herself (Vacc et al., 2003). Cultural awareness is also a capability to realize a culture’s impacts on human behavior and values (Wunderle, 2006). In creating cultural products, the attractiveness of content and the inherent culture of the producer are developed spontaneously. The producer side seems to be more influential in cultural transmission (Morgan and Shanahan, 1997). The producer’s experience, emotional engagement, and cultural viewpoint are ingrained in the generation process of content creation (Wang, 2020). By being exposed to information regarding dissimilar cultures through cultural products such as TV series, learners develop awareness and make critical judgments regarding their beliefs, practices, and perspectives by comparing them with those of other cultures (Yanhao and Mustafa, 2021). As different aspects of culture are transferred from the content of a story (Pacheco, 2020) we suggest the following:

*H3a*: TV series consumption positively influences cultural awareness.

### TV series consumption and cultural acceptance

Cultural acceptance is defined as the embrace of other cultures or others’ cultural identities. Kwon (2015) stated that culture acceptance is the willingness to accept or try something unfamiliar, such as ethnic food from outside one’s group. Similarity is very crucial for consumers’ acceptance of new culture (Rentfrow et al., 2011). To realize effective communication, communicators intend to enhance the conformity of communication between audiences (Gevorgyan and Manucharova, 2015; Gao et al., 2020). In the case of global brand digital marketing, Almeida (2015) found that successful marketers understand cultures deeply. The notion of similarity in communication could increase consumer acceptance and purchasing intent (Akdeniz and Talay, 2013; Hersleth et al., 2015). As per Lee (2019), “Film has a long history of not only entertaining but also educating, breaking stereotypes, and transcending borders at different levels to foster mutual understanding through the exchange of ideas, information, art, and other aspects of culture among nations and their peoples.”

Therefore, we hypothesize the following:

*H3b*: TV series consumption positively influences cultural acceptance.

### TV series consumption and involvement with series

Involvement constitutes how deeply consumers become engaged with cultural products. Higher levels of involvement are supposed to attract and encourage the participation of consumers. As television watching could be triggered by the desire to socialize with others (Rubin, 1983), consumers can also participate and interact with other users through media consumption, which could bring socializing gratifications in media usage (Apaolaza et al., 2015). Unlike traditional consumers, modern consumers of cultural products who build fan communities on online platforms (Page and Thomas, 2011) are not passive audiences as they could influence producers and production processes (Pearson, 2010). Fan communities are assembled according to similar preferences for cultural patterns (Mulyana et al., 2019). Community members like to discuss their interests and idols with other fans *via* the internet (Jenkins, 2007). Fandom plays a crucial role between producers and consumers as fans tend to be heavily engaged (Fiske, 2012). The greater the fan interaction, the higher the level of involvement expected. Consequently, the consumption of cultural products would affect consumers’ involvement with series.

Thus, we suggest the following:

*H3c*: TV series consumption positively influences involvement with series.

### Cultural awareness and cultural acceptance

Cultural acceptance is the way consumers embrace a new culture. Cultural products are not limited to amusement as they involve performing or acting and are constantly present (Hall, 1992). As producers try to increase communication (Gevorgyan and Manucharova, 2015), consumers accordingly perceive a culture’s identity in their consumption (Satrio et al., 2020). Media consumption’s cultivation effect also influences viewers’ worldview (Gerbner et al., 2002). With the aspects of new media (e.g., video on demand), Steiner and Xu (2018) stated that cultural integration is a motivation for continued viewing (binge watching) habits. Consumer acceptance could also come in the form of purchase intent (Hersleth et al., 2015), which is a challenging issue for digital marketers (Almeida, 2015). National representations of history, heritage, myths, and folklores that comprise the cultural foundation can also be used as national resources, particularly in the technological era where diverse international products are available at a click. Perception as the first stage of cultural product consumption could affect cultural acceptance. As cultural awareness is the antecedent to the decision to learn more and exchange cultural background knowledge, spectators’ awareness of depicted culture arguably leads to effective acceptance.

Therefore, the following hypothesis is proposed:

*H4*: Cultural awareness positively influences cultural acceptance.

### Involvement with series and cultural acceptance

When engaging in cultural branding through films, production must consider the complex and unforeseeable nature of associations that overseas audiences will form. Goffman (1974) identified that engagement relates to emotional reactions resulting from social interactions, whereas marketing specialists consider it as an indicator of the level of intention (Scott and Craig-Lees, 2010). Consumers absorb cultural context from media and become engaged during consumption. Cross-cultural entertainment fosters lasting bonds between viewers and characters from foreign cultures including purchase intention (Adamczyk, 2017; Sun et al., 2022). Furthermore, the media aids in the formation of social and communication networks among fandoms and diverse social structures. Audiences are also characterized as keen readers in web novel communities, story co-producers (Grey, 2010), and participants willingly aiding media distribution to a broader group of consumers. Socialization and interaction also bring user-generated content to enhance involvement (Gilardi et al., 2018). Consumers of cultural products seem to trust user-generated online reviews more than other sources (Camacho-Otero et al., 2019). Mulyana et al. (2019) found that active fans drive others to support their favorite products through merchandise (e.g., bags and clothes). This reflects the increased knowledge intent concerning foreign culture resulting from cultural product consumption.

Therefore, we infer that involvement with cultural products drives consumers to embrace foreign culture and propose the following:

*H5*: Involvement with series positively influences cultural acceptance.

### Cultural awareness and cultural knowledge

Culture learning is defined as a cultural adaptation process that is free from significant psychological strain on learners and is considered a facilitator of cultural appropriation (Lefdahl-Davis and Perrone-McGovern, 2015; Belford, 2017). Psychological distress emerges when individuals are culture shocked after entry into a foreign environment without a prior adjustment period. Culture learning acts as a buffer and facilitator of intercultural transmission (Pacheco, 2020), which is often cultivated through media content, whereby learners are primed to a second culture through familiarization with content that evokes positive associations related to a foreign culture (e.g., norms, values, and beliefs; Mahmood, 2014). Cultural knowledge is characterized by the understanding and mindfulness of social groups, products, and practices. As cross-cultural knowledge is mainly relational and social (Elola and Oskoz, 2008), it concerns the awareness and perceptions of the interlocutor’s country and cognizance of the general process of societal interaction.

The knowledge of other cultures is a product of not only one-sided media portrayal but also socialization and cultural learning through online interaction (Elola and Oskoz, 2008). Media cultural products facilitate the dissemination of values and ideas, thus raising cultural awareness and extending cultural knowledge among foreign consumers. Such strategy of creating fandom communities based on domestic characters and myths encourages international communication. Cultural knowledge from cultural product consumption could vary with consumers’ psychological aspects (Khan et al., 2012; Senivongse and Bennet, 2022). Additionally, consumers can now learn about traditional cultures and gain awareness through advanced information technology (Huang et al., 2015; Siddharthan and Narayanan, 2018).

We infer that consumers’ higher cultural awareness could increase knowledge and understanding. Consequently, we suggest the following:

*H6*: Cultural awareness positively influences cultural knowledge.

### Cultural acceptance and cultural knowledge

Cultural acceptance could be in the form of cultural hybridization (Pieterse, 2009), particularly in cultural products. Kogut and Singh (1988) found that cultural product consumption and acceptance reduces the cultural gap between countries. For instance, through K-pop culture, overseas fans often consume Korean fashion and beauty products and even travel to Korea (Kwak et al., 2019). Consumers easily respond to cultural products with broader cultural acceptance (Moon et al., 2016). Having profound cultural knowledge ensures the successful dissemination of cultural products. Akdeniz and Talay (2013) identified that cultural variation is due to influential factors (e.g., actors’ persona and presence) in movie sales. Titling and movie synchronization to other languages must be comprehensive to achieve natural sounds and accurately convey meaning (Gao et al., 2020). Moreover, consumers often look for content that matches their circumstances, culture, characteristics, and preferences (Rentfrow et al., 2011). Following the definitions and impact of variables and conceptualized relationships, we conclude that cultural knowledge demonstrates a higher degree of cultural engagement. Cultural knowledge consists of cultural comprehension and leads to cross-cultural appropriation (Elola and Oskoz, 2008).

Therefore, we suggest the following:

*H7*: Cultural acceptance positively influences cultural knowledge.

### Involvement with series and cultural knowledge

Cultural product consumers trust user-generated online reviews more than other sources (Camacho-Otero et al., 2019). Trust among consumers has been observed in media cultural products such as cultural blogs (Magno, 2017), online literature (Tian and Adorjan, 2016), and webtoon and web novel communities (Shim et al., 2020). Readers create communities to share and exchange information and achieve a common understanding of cultural products (Tian and Adorjan, 2016). The fandom of readers can be considered a vital transmitter in expanding information and knowledge (Dwyer, 2019). Their base of trust is important in the percentage of the global gross domestic product that creative and cultural industries generate annually as they are willing to translate and share content with community members voluntarily (Shim et al., 2020). They would have the deep relation between their feelings and commitment with their involved brand (Bian and Yan, 2022). With a higher level of involvement, consumers voluntarily share their knowledge and information with community members.

Consequently, we hypothesize the following:

*H8*: Involvement with series positively influences cultural knowledge.

**Figure 1:**
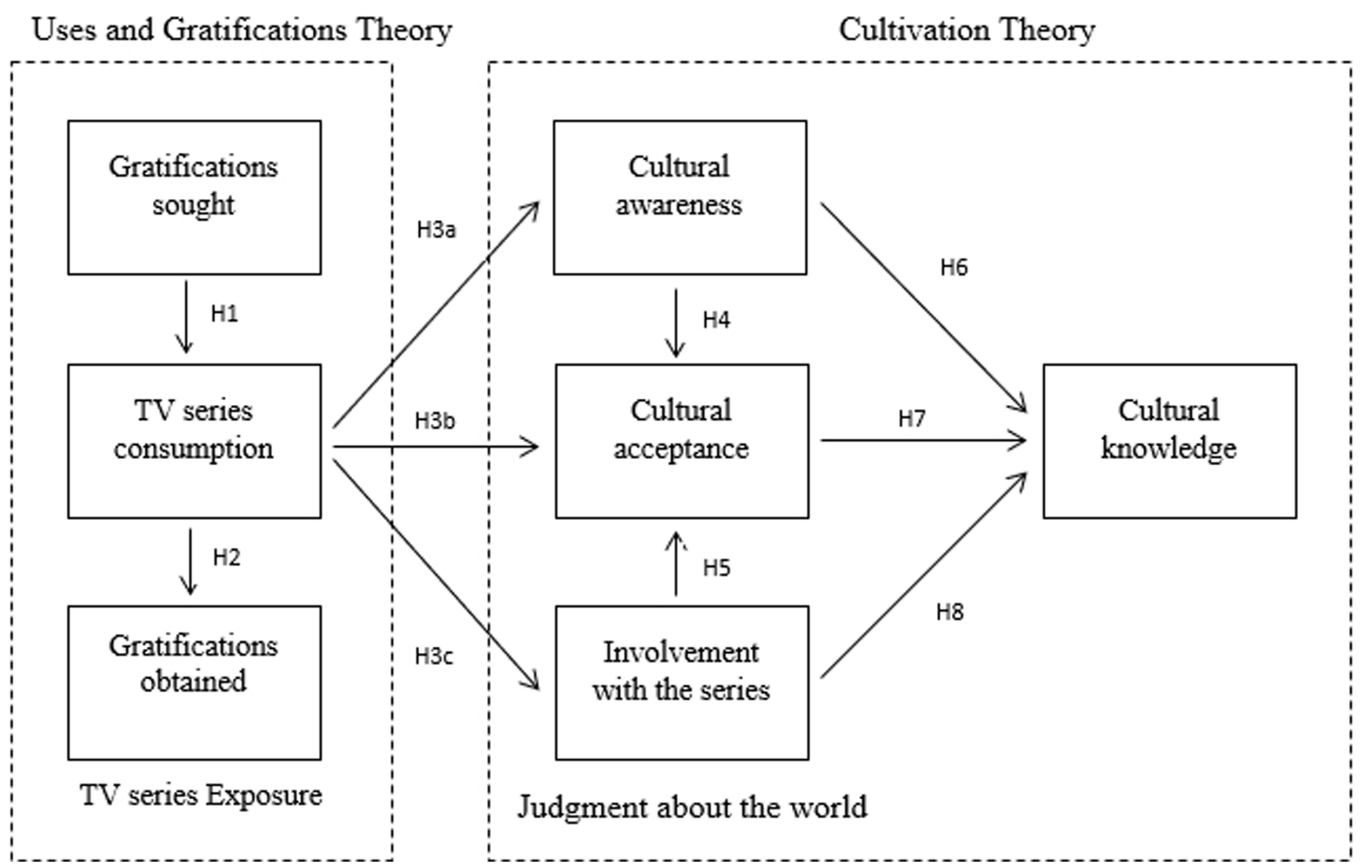
Research model of the study.

The research model is illustrated in Figure 1.

## Research methodology

### Measurement and data collection

A survey through online questionnaire was conducted to evaluate the respondents’ attitudes and behavior and illustrate different scenarios of how the respondents feel, think, or behave. The variables included the gratification sought, Chinese TV series consumption, the gratification obtained, cultural awareness, cultural acceptance, involvement with series, and cultural knowledge. The 5-point Likert scale ranging from strongly agree (5) to strongly disagree (1) was used as a measurement tool.

The measurement questions of each variable were formulated by adopting keywords and concepts from previous studies. Each variable consisted of five item questions for measuring the respondents’ scenarios.

The study’s target population consisted of Thai netizens who watch Chinese TV series. As the population is unknown, estimation by samples was used. Purposive voluntary sampling was conducted to accumulate data from the participants. The online questionnaire was back-translated. The questionnaire in the Thai language was posted on the public social media websites of Chinese TV series lovers in Thailand. The data collection was anonymous and was conducted between October and December 2021. There were 856 respondents included in the sample for analysis. Young adults or older first-jobbers aged 26–30 years comprised the largest percentage of respondents (78%; Wisetwongchai, 2020). The mean of all respondents’ ages was 25.27 years. Moreover, there was a higher proportion of female viewers (90%) than male viewers. More than half of respondents (497 or 58.1%) spent between 0 and10 hours per week watching series.

To confirm the reliability of the measurement tool, we calculated the Cronbach’s alpha for each scale (Table 1). The cultural acceptance scale had the highest Cronbach’s alpha of 0.895, followed by cultural knowledge and level of TV series consumption at 0.888 and 0.874, respectively. The Cronbach’s alpha of the gratification sought scale was 0.772. As all the values were higher than 0.7, the instrument was found to have sufficient reliability.

**Table 1:** Measurement and reliability test.

Variable	Cronbach’s Alpha	N of Items	Reference(s)
Gratification sought	0.772	5	Papacharissi (2009a)
TV series consumption	0.874	5	Huang et al. (2015)
Gratification obtained	0.838	5	Stafford et al. (2004)
Cultural awareness	0.856	5	Vacc et al. (2003)
Involvement with series	0.821	5	Kwak et al. (2019), Moon et al. (2016)
Cultural acceptance	0.895	5	Gilardi et al. (2018), Tian and Adorjan (2016)
Cultural knowledge	0.888	5	Elola and Oskoz (2008)

## Statistical analysis and results

Statistical analysis was conducted using STATA version 16 MP. As part of the statistical analysis, we calculated descriptive statistics, reliability, correlation analysis, confirmatory factor analysis (CFA), and structural model testing.

### Correlation analysis

Correlation analysis was performed to indicate appropriate correlation (Table 2). All correlation coefficients showed a moderate relationship level ranging from 0.4 to 0.8. Thus, we confidently included fitting factors in our research model. The highest correlation coefficient among all pairs of analysis was 0.745, which was the correlation coefficient between cultural acceptance and cultural knowledge. Variance of inflation factor (VIF) was calculated to test for multicollinearity. All correlation values showed no serious multicollinearity problem; the highest and lowest VIF values were less than 10 at 3.009 and 1.927, respectively (Schober et al., 2018).

**Table 2:** Correlation analysis.

Variables	GraSoug	SeriCons	GraObt	CulAwar	InvFeel	CulAcc	CulKnow	VIF
GraSoug	1							2.103
	$\bar{x}$ = 4.50							
SeriCons	0.491^**^	1						1.990
	*p* < 0.01	$\bar{x}$ = 3.94						
GraObt	0.714^**^	0.648^**^	1					3.009
	*p* < 0. 01	*p* < 0.01	$\bar{x}$ = 4.30					
CulAwar	0.536^**^	0.486^**^	0.627^**^	1				1.927
	*p* < 0.01	*p* < 0.01	*p* < 0.01	$\bar{x}$ = 4.27				
InvFeel	0.427^**^	0.557^**^	0.529^**^	0.559^**^	1			2.120
	*p* < 0.01	*p* < 0.01	*p* < 0.01	*p* < 0.01	$\bar{x}$ = 3.62			
CulAcc	0.435^**^	0.538^**^	0.524^**^	0.509^**^	0.655^**^	1		1.980
	*p* < 0.01	*p* < 0.01	*p* < 0.01	*p* < 0.01	*p* < 0.01	$\bar{x}$ = 4.03		
CulKnow	0.443^**^	0.509^**^	0.547^**^	0.577^**^	0.611^**^	0.745^**^	1	
	*p* < 0.01	*p* < 0.01	*p* < 0.01	*p* < 0.01	*p* < 0.01	*p* < 0.01	$\bar{x}$ = 3.95	

** Correlation is significant at the 0.01 level (two-tailed).

### Confirmatory factor analysis (CFA)

CFA was performed, and the degree of model fit is shown in Table 3. Hair et al. (2014) recommended that a fit model should have normed chi-square values less than 3, root mean square error of approximation (RMSEA) less than 0.08, standardized root mean squared residual (SRMR) less than 0.08, and comparative fit index (CFI) higher than 0.90; values higher than 0.95 indicate excellent fitness.

**Table 3:** Degree of model fit for CFA.

Model analyzed	Chi-square (χ2)	χ2/df	value of *p*	RMSEA	SRMR	CFI
Confirmatory factor analysis (CFA)	1829.126	3.425	*p* < 0.01	0.053	0.053	0.930
Criteria (Bentler, 1990; Steiger, 2007; Hair et al., 2014)	–	<3	–	<0.06	<0.06	>0.90/ 0.95

Table 3 shows that the RMSEA is less than 0.80 at 0.053, the SRMR is also less than 0.80 at 0.053, and the CFI is higher than 0.90 at 0.930, thereby indicating good model fit (Bentler, 1990; Byrne, 2010). Only the normed Chi-square exceeds 3 (χ^2/df = 3.425). Nevertheless, chi-square is very sensitive to large sample sizes and could decrease the value of p. Excessive emphasis on chi-square might lead to a preference for the null hypothesis not being rejected (Alavi et al., 2020).

The factor loadings of each variable item scale are shown in Table 4. All factor loadings exceed 0.55, as suggested by Fornell and Larcker (1981). Furthermore, all items are statistically significant at the 0.01 level.

**Table 4:** Factor loadings.

Factors of variables	Coef.	S.E.	z	P
GraSoug1←GraSoug	1	–	–	
GraSoug2←GraSoug	1.164	0.071	16.31	**
GraSoug3←GraSoug	1.176	0.069	17.09	**
GraSoug4←GraSoug	1.081	0.067	16.04	**
GraSoug5←GraSoug	1.007	0.062	16.19	**
SeriCons1←SeriCons	1	–	–	**
SeriCons2←SeriCons	0.787	0.036	21.86	**
SeriCons3←SeriCons	0.958	0.033	28.61	**
SeriCons4←SeriCons	0.936	0.041	23.04	**
SeriCons5←SeriCons	0.863	0.035	24.76	**
GraObt1←GraObt	1	–	–	**
GraObt2←GraObt	1.134	0.040	28.15	**
GraObt3←GraObt	1.020	0.039	26.20	**
GraObt4←GraObt	0.883	0.034	25.83	**
GraObt5←GraObt	0.627	0.049	12.70	**
CulAwar1←CulAwar	1	–	–	**
CulAwar2←CulAwar	1.096	0.046	23.66	**
CulAwar3←CulAwar	0.966	0.040	23.91	**
CulAwar4←CulAwar	0.857	0.043	19.95	**
CulAwar5←CulAwar	1.036	0.051	20.31	**
InvlSeri 1← InvlSeri	1	–	–	**
InvlSeri 2← InvlSeri	1.621	0.096	16.81	**
InvlSeri 3← InvlSeri	1.683	0.097	17.31	**
InvlSeri 4← InvlSeri	1.731	0.123	14.02	**
InvlSeri 5← InvlSeri	1.650	0.102	16.05	**
CulAcc1←CulAcc	1	–	–	**
CulAcc2←CulAcc	0.929	0.038	24.32	**
CulAcc3←CulAcc	0.979	0.039	24.57	**
CulAcc4←CulAcc	0.859	0.036	24.04	**
CulAcc5←CulAcc	0.977	0.037	26.04	**
CulKnow1←CulKnow	1	–	–	**
CulKnow2←CulKnow	0.987	0.028	34.71	**
CulKnow3←CulKnow	0.951	0.028	34.22	**
CulKnow4←CulKnow	0.917	0.045	20.22	**
CulKnow5←CulKnow	0.837	0.040	20.88	**

** Correlation is significant at the 0.01 level (two-tailed).

**Figure 2:**
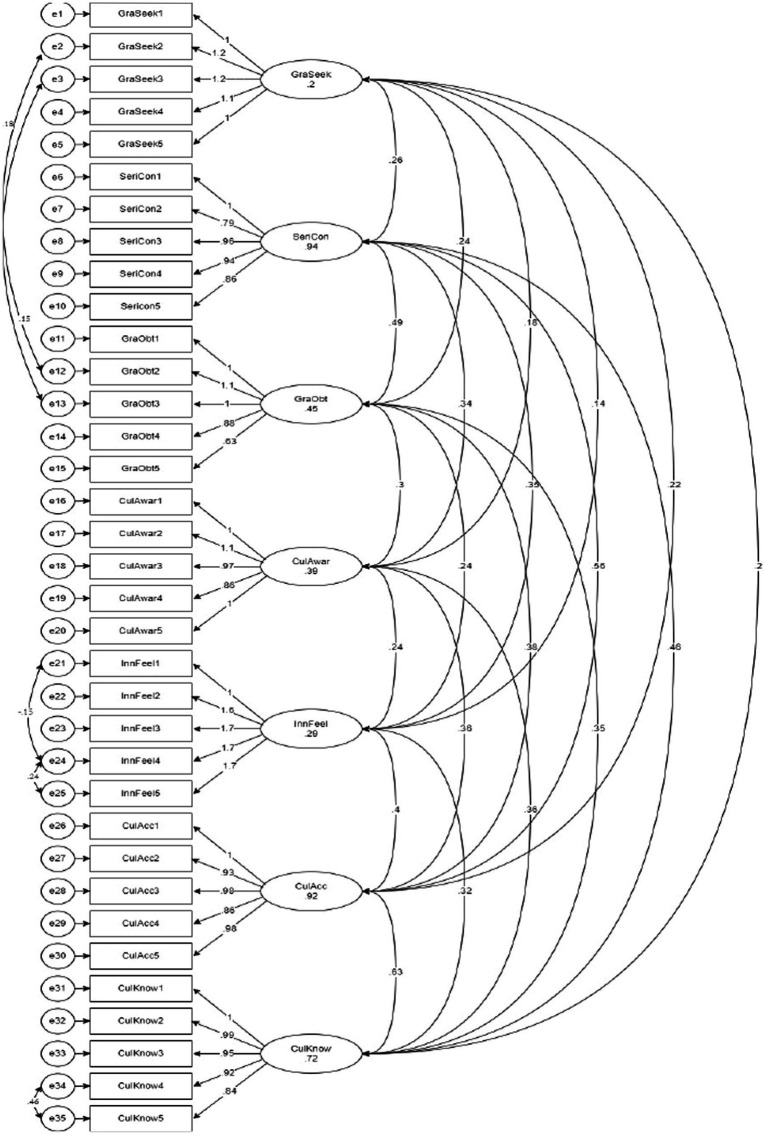
Confirmatory factor analysis.

The CFA for each variable, along with the item scales, is shown in Figure 2.

### Structural equation model (SEM) testing

The model was tested using a structural equation model (SEM) and path analysis. The degree of model fit for the structural model was calculated to confirm that the structural model is consistent with the theoretical model. In Table 5, we compare the hypothesized model with a modified SEM.

**Table 5:** Degree of Model Fitness Statistics.

Model analyzed	Chi-square (χ2)	value of *p*	RMSEA	SRMR	CFI
Hypothesized model	787.861	0.000	0.287	0.157	0.772
Modified structural equation model	0.267	0.605	0.000	0.002	1.000
Criteria (Diamantopoulos and Siguaw, 2000; Schumacker and Lomax, 2010)	–	>0.05	<0.05	<0.06	>0.90/0.95

The degree of model fitness illustrates an adequate fit. The chi-square (χ^2) value is 0.267 (χ^2 = 0.267), indicating the model’s likelihood ratio test. Fit is achieved when the value of p is higher than 0.05 (Schumacker and Lomax, 2010). The value of p of 0.605 indicates appropriate model fit. The RMSEA of the analysis should not exceed 0.05 (Diamantopoulos and Siguaw, 2000). Similarly, the SRMR should not exceed 0.05. Finally, the CFI value should be higher than 0.90–0.95 to define model fitness; values higher than 0.95–1.00 indicate good fitness (Schumacker and Lomax, 2010). All the indices are of appropriate values, thus indicating a fit (Table 5).

The research model was modified to show acceptable model fit. A path from gratification obtained to cultural knowledge was added. The hypothesis testing indicated that H1 is accepted, with the coefficient value (B) and value of p being 0.850 and less than 0.01, respectively. This result confirms that gratification sought has a positive impact on Chinese TV series consumption (B = 0.850, value of *p* < 0.01). H2 is also accepted, with the coefficient value (B) and value of *p*  being 1.041 and less than 0.01, respectively (B = 1.041, value of *p* < 0.01). This result confirms that Chinese TV series consumption has a positive impact on the gratification obtained. H3a is accepted, with the coefficient value (B) and value of p being 0.775 and less than 0.01, respectively (β = 0.775, value of *p* < 0.01). This result confirms that Chinese TV series consumption has a positive impact on cultural awareness. H3b is also accepted, with the coefficient value and value of p being 4.923 and less than 0.01, respectively (B = 4.923, value of *p* = < 0.01). This result confirms that Chinese TV series consumption has a positive impact on cultural acceptance. H3c is accepted, with the coefficient value and value of p being 0.867 and less than 0.01, respectively (B = 0.867, value of *p* < 0.01). Hence, Chinese TV series consumption has a positive impact on involvement with series. Meanwhile, H4 is also accepted, with the coefficient value (B) and value of p being 12.606 and less than 0.01, respectively (B = 12.606, z = 4.42, value of *p* < 0.01). The result indicates that cultural awareness has a positive impact on cultural acceptance. H5 is rejected as the coefficient value (B) and value of *p* are −15.900 and less than 0.01, respectively (B = −15.900, value of *p* < 0.01). This result shows that involvement with series has a positive impact on cultural acceptance. As for H6, it is accepted, with the coefficient value (B) and value of p being 0.242 and less than 0.01, respectively (B = 0.242, value of *p* < 0.01). This result confirms that cultural acceptance has a positive impact on cultural knowledge. H7 is accepted, with the coefficient value (B) and value of *p* being 0.576 and less than 0.01, respectively (B = 0.576, value of *p* < 0.01). This result shows that cultural acceptance has a positive impact on cultural knowledge. Finally, H8) is rejected, with the coefficient value and value of p being 0.050 and 0.552, respectively (B = 0.050, value of *p* = 0.552). This result confirms that involvement with series positively impacts cultural knowledge but is not statistically significant (Table 6).

**Table 6:** Regression weights, errors, and *p*-values.

Relationships assessed	Coef. (B)	Std.err	z	95% CI	
Gratification Sought→Series Consumption	0.850**	0.052	16.50	0.749	0.951
Series Consumption→ Gratification Obtained	1.041**	0.055	18.84	0.932	1.149
Series Consumption→Cultural Awareness	0.775**	0.052	14.79	0.672	0.878
Series Consumption→Cultural Acceptance	4.923**	1.224	4.02	2.524	7.323
Series Consumption→Involvement	0.867**	0.055	15.91	0.760	0.974
Cultural Awareness→Cultural Acceptance	12.606**	2.850	4.42	7.020	18.192
Involvement→Cultural Acceptance	−15.900**	3.506	−4.53	−22.724	−9.028
Gratification Obtained→Cultural Knowledge	0.085	0.063	1.34	−0.039	0.209
Cultural Awareness→Cultural Knowledge	0.242**	0.038	6.40	0.168	0.317
Cultural Acceptance→Cultural Knowledge	0.576**	0.149	3.86	0.283	0.868
Involvement→Cultural Knowledge	0.050	0.084	0.060	−0.115	0.215

** A coefficient is significant at the 0.01 level (two-tailed).

**Figure 3:**
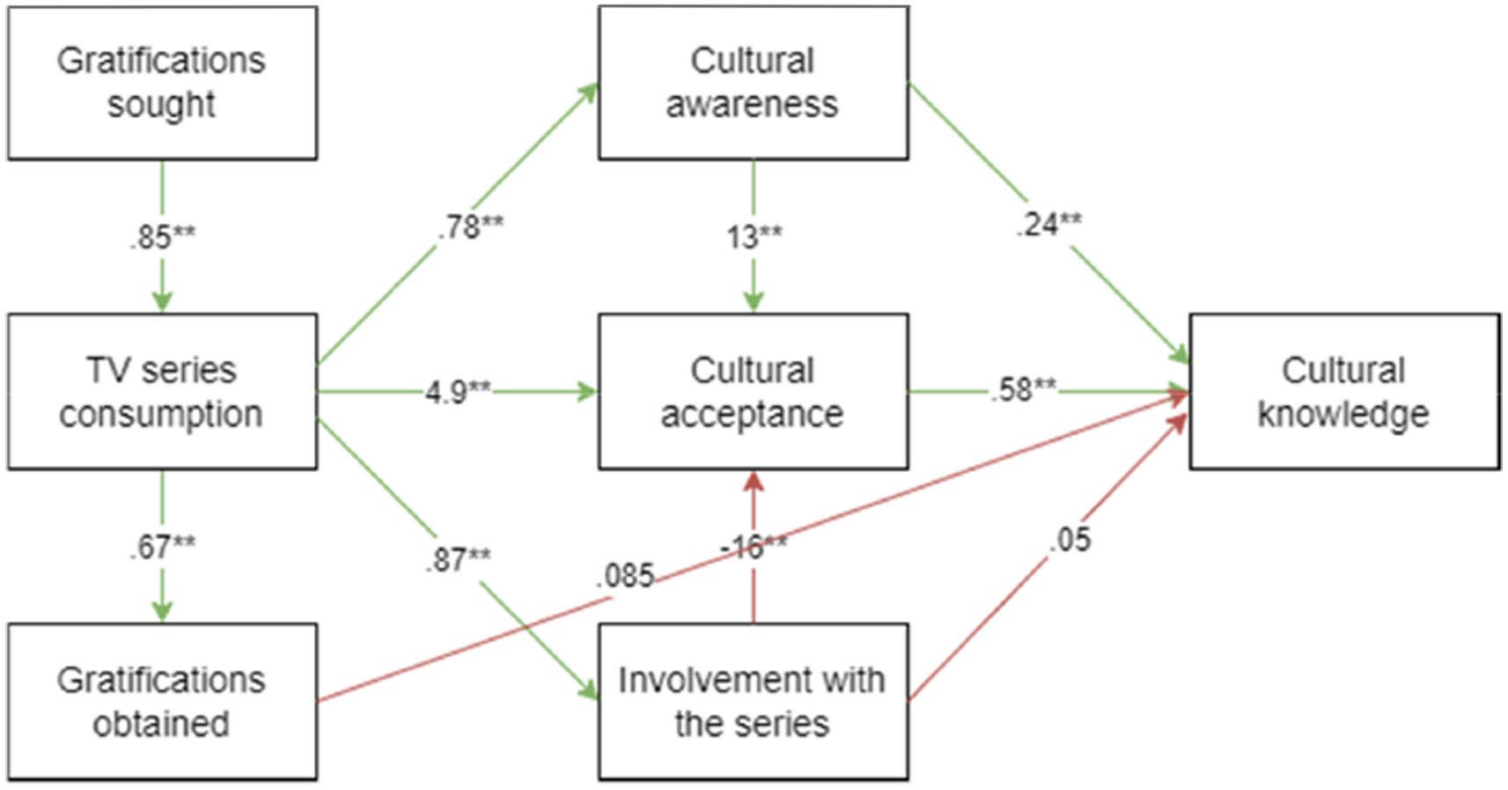
Results of research model. ** is 0.01 level of significance.

The path analysis in Table 6 is shown as a research model in Figure 3. The decisions on research hypotheses are displayed in Table 7.

**Table 7:** Summary of research hypotheses.

Hypotheses	Decision
H1: Gratification sought positively influences TV series consumption.	Accepted
H2: TV series consumption positively influences the gratification obtained.	Accepted
H3a: TV series consumption positively influences cultural awareness.	Accepted
H3b: TV series consumption positively influences cultural acceptance.	Accepted
H3c: TV series consumption positively influences involvement with series.	Accepted
H4: Cultural awareness positively influences cultural acceptance.	Accepted
H5: Involvement with series positively influences cultural acceptance.	Rejected
H6: Cultural awareness positively influences cultural knowledge.	Accepted
H7: Cultural acceptance positively influences cultural knowledge.	Accepted
H8: Involvement with series positively influences cultural knowledge.	Rejected

## Discussion

The basic conceptual framework of this research was confirmed, that is, there are cultivation impacts from media usage initiated by gratification sought or motivation for gratification in media exposure. Intercultural engagement is fostered through sharing of immersive content using affordable technologies (Siddharthan and Narayanan, 2018), in which cultural learning and intercultural adaptation can spontaneously occur by simply watching foreign movies and TV series (Li and Chen, 2014; Pacheco, 2020). Contemporary technology, popular social platforms, and interactive features have domesticated the experience of cultural learning while eliminating culture shocks by fostering communication and engagement with other cultures, encouraging the exchange of intercultural norms, and promoting immersion in other lifestyles and thinking patterns (Pacheco, 2020). Herein, we focused only on Chinese TV series watching as a form of cultural product consumption. The findings of the study correspond to those of the study of Bilandzic and Rössler (2004). Chinese TV series consumption could provide satisfaction and fulfill consumers’ gratification need quite well because series watching could significantly respond to gratification sought and gratification obtained. The findings correspond to those of previous studies on U&G by Dhir et al. (2017) and Papacharissi (2009a).

Furthermore, we combine two prominent theoretical frameworks to explore the educational prospect of audiovisual content in forming a perception of foreign culture’ traditions, habits, norms and values and gaining an understanding of inherent lifestyles, behavioral patterns, heritage and ethnicity. Our endeavor concerns itself with the investigation and analysis of theoretical applicability in the case of China-Thailand contemporary media culture. To that end, we provide a literature review and research results of numerous prior inquiries from media and communication, marketing and ethnology studies. Furthermore, our results on the effects of cinema and digital streaming provide confirmation for the positive mutual association between the Gratification and Cultivation theories, and their applicability in the current context. An important contribution of the study is that the need for gratification significantly and positively impacts consumption and cross-cultural learning and raises cross-cultural awareness. However, personal engagement, i.e., inner experiences, contrary to our assumptions, do not influence cultural knowledge and acceptance.

The empirical study showed that Chinese TV series consumption affects the cultural awareness, cultural acceptance, and cultural knowledxx`x`ge of Thai viewers. For cultural awareness, this research confirmed that consumers perceive and acknowledge cultural differences from what they experience in TV series. This finding corresponds to the study of Ferguson (2008), which found that people can identify their perceptions from media usage. Chinese TV series consumption also influenced cultural acceptance, indicating that consumers not only acknowledge the differences between new and inherent cultures but also accept cultures portrayed in the series. This result corresponds to those in the study of Hersleth et al. (2015) and Almeida (2015), who revealed that the distribution of media content should come with cultural acceptance among consumers. In other words, letting consumers accept culture is another crucial function of media. Additionally, cultural awareness and acceptance influence cultural knowledge due to Chinese TV series viewing. The participants reported that they could better understand Chinese culture, which are not only the external cultures that could be seen, but also the internal ones, including beliefs, attitudes, norms, and values. Corresponding to the study of Machová et al. (2022) found that cultural, social and personal factors (e.g., gender, reference group, social class, religious and ethnical group and the age of the customer) greatly influence consumer habits. These are the external factors, while the internal factors are psychological factors—including the following: motivation, perception, learning and attitude.

One variable that conflicted with previous studies is the involvement in series. This variable was measured by the respondents’ involvement with TV series, which could be in different forms or behaviors. Some consumers may be active fans who follow and support their favorite stars or series. Some consumers might prefer to socialize or interact with other viewers on social networking platforms, and some may contribute to their favorite series by encouraging others to watch the series. All these behaviors were found as significant factors in how consumers deal or interact with the consumption of cultural products.

The aforementioned additional path for model modification also plays a vital role among this cultural product consumption of individuals. Although the path shows insignificant value, it makes the model fit with theoretical concept. This finding brings us to a deeper question of future study regarding theoretical model in new media environment. Since the cultural knowledge is considered as the final outcome of cultural learning from cultivation effect, similarly gratification obtained is regarded as the final outcome of U&G or the satisfaction of media consumption. Consequently, this result implies us that individual could learn or gain knowledge and gratification simultaneously, by having media and communication as functional instrument (Tirasawasdichai and Pookayaporn, 2021).

This study is valuable as it provides a contemporary reinterpretation of cultural learning, adaptation, and cross-cultural awareness in media psychology, as experienced by present-day foreign TV series consumers and accounting for the significant role of modern technology as the driver of cultural acceptance. The main contribution of the study is the critical review of the main yet underutilized media theories in the present-day digital global environment. The study provides an illuminating discussion on sociocultural acclimation and appropriation as an addition to scholarly thought regarding immersive mediated experiences causing cross-cultural knowledge diffusion. Our objective is to revitalize the discourse on the cultivation effect and transitional experiences as antecedents of affective culture learning perpetuating cultural branding. Our considerations are in line with modern experiences and current sociopsychological insights. This study also innovates by combining the U&G and cultivation theories; this combination is rare despite the two being core theories in the media and communication academic field. Moreover, the theories have been validated in the context of digital and online channels. The findings herein also shed light on sustainable international communication policymaking and the construction of a country’s image. They may also help producers design their products and market them internationally. Undeniably, cultural products, regarded as a part of soft power, are powerful as they drive economies and influence consumers’ attitudes and values (Rabêlo Neto et al., 2019). Additionally, this study specified the current context of online and digital environment that consumers could access quite freely. Moreover, with the diversity of media channels in this era, the empirical results of this study could explain well that whether the impacts of cultivation effect from tv viewing are still existing among online and digital environment of media.

### Limitations and future directions

The study suffers from usual limitations, which should be addressed in future studies. For example, the data were collected using self-reported measures. Additionally, the sample was heterogeneous given that internet users in Thailand cover a wide range of groups or segments. Moreover, each generation’s proportion or ratio of internet usage might differ, thus restricting the identification of segments or groups that best represent internet users. Another important limitation of the study is the accessibility of consumers to Chinese TV series in the current context. Nowadays, consumers can conveniently access media content *via* many channels or devices. Therefore, different channels could provide different experiences in consumption of media, especially TV series or serial video content (Anghelcev et al., 2020). Future studies should investigate the effects of media channels as media plays a crucial role in consumer experience. Additionally, specific population segments and types of series should be investigated in the future. Future studies could also assess cultural acceptance as an outcome of cultural awareness and knowledge given that prior studies have also suggested such relationship.

## Data availability statement

The raw data supporting the conclusions of this article will be made available by the authors, without undue reservation.

## Ethics statement

The studies involving human participants were reviewed and approved by the study was conducted according to the guidelines of the Declaration of Helsinki, and approved by the Institutional Review Board of Shanghai Jiao Tong University (IRB# 86413009). The patients/participants provided their written informed consent to participate in this study.

## Author contributions

TT: data curation and resources. TT and HA: formal analysis and methodology. TT and BO: investigation and writing – original draft. BO: project administration. TT, BO, and HA: writing – review and editing. All authors contributed to the article and approved the submitted version.

## Conflict of interest

The authors declare that the research was conducted in the absence of any commercial or financial relationships that could be construed as a potential conflict of interest.

## Publisher’s note

All claims expressed in this article are solely those of the authors and do not necessarily represent those of their affiliated organizations, or those of the publisher, the editors and the reviewers. Any product that may be evaluated in this article, or claim that may be made by its manufacturer, is not guaranteed or endorsed by the publisher.
